# MALAT-1 Is a Key Regulator of Epithelial–Mesenchymal Transition in Cancer: A Potential Therapeutic Target for Metastasis

**DOI:** 10.3390/cancers16010234

**Published:** 2024-01-04

**Authors:** Mohamed Ali Hussein, Kamyab Valinezhad, Eman Adel, Gnanasekar Munirathinam

**Affiliations:** 1 Department of Pharmaceutical Services, Children’s Cancer Hospital Egypt, Cairo 57357, Egypt; mohamedalihussien8554@gmail.com; 2 Department of Biology, School of Sciences and Engineering, American University in Cairo, New Cairo 11835, Egypt; emanadel@aucegypt.edu; 3 Department of Biomedical Sciences, College of Medicine, University of Illinois, Rockford, IL 61107, USA; kvalin2@uic.edu

**Keywords:** MALAT-1, EMT, metastasis, chemoresistance, microRNA, lncRNA

## Abstract

**Simple Summary:**

Metastasis-associated lung adenocarcinoma transcript-1 (MALAT-1) is overexpressed in several cancers and exerts its effect by controlling gene expression and stimulating cell proliferation, migration, and metastasis and playing a dynamic role in mediating the epithelial-to-mesenchymal transition (EMT), which leads to the acquisition of stem cell-like properties and chemoresistance. MALAT-1 modulates EMT by interacting with various intracellular signaling pathways, including phosphoinositide 3-kinase (PI3K)/Akt and Wnt/β-catenin. It also sponges microRNAs, consequently increasing the gene expression of several essential genes regulating cancer progression and metastasis, rendering it a good candidate for therapeutic intervention. Several innovative approaches have been exploited to target MALAT-1, such as short hairpin RNAs (shRNAs), antisense oligonucleotides (ASOs), and natural products.

**Abstract:**

Metastasis-associated lung adenocarcinoma transcript-1 (MALAT-1) is a long intergenic non-coding RNA (lncRNA) located on chr11q13. It is overexpressed in several cancers and controls gene expression through chromatin modification, transcriptional regulation, and post-transcriptional regulation. Importantly, MALAT-1 stimulates cell proliferation, migration, and metastasis and serves a vital role in driving the epithelial-to-mesenchymal transition (EMT), subsequently acquiring cancer stem cell-like properties and developing drug resistance. MALAT-1 modulates EMT by interacting with various intracellular signaling pathways, notably the phosphoinositide 3-kinase (PI3K)/Akt and Wnt/β-catenin pathways. It also behaves like a sponge for microRNAs, preventing their interaction with target genes and promoting EMT. In addition, we have used bioinformatics online tools to highlight the disparities in the expression of MALAT-1 between normal and cancer samples using data from The Cancer Genome Atlas (TCGA). Furthermore, the intricate interplay of MALAT-1 with several essential targets of cancer progression and metastasis renders it a good candidate for therapeutic interventions. Several innovative approaches have been exploited to target MALAT-1, such as short hairpin RNAs (shRNAs), antisense oligonucleotides (ASOs), and natural products. This review emphasizes the interplay between MALAT-1 and EMT in modulating cancer metastasis, stemness, and chemoresistance in different cancers.

## 1. Introduction

**Figure 1:**
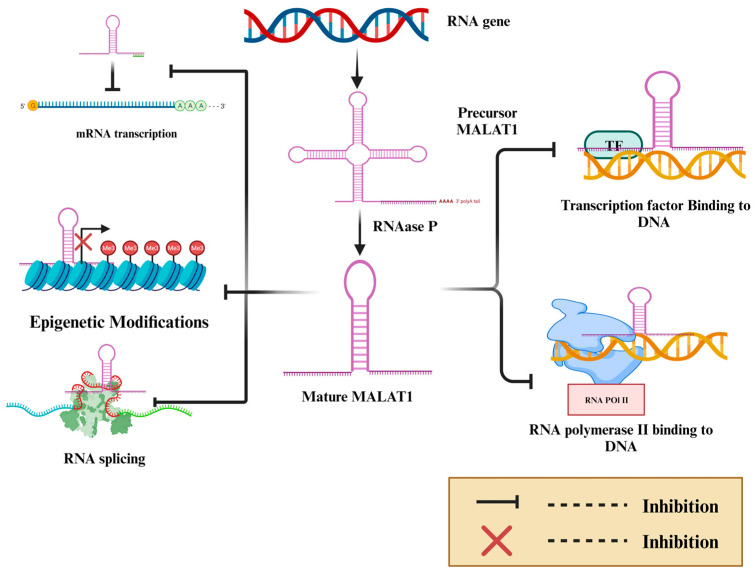
MALAT-1 is an RNA gene encoded by chromosome 11q13. MALAT-1 is initially transcribed as a precursor immature transcript, which the enzyme RNAase P processes to produce mature MALAT-1. MALAT-1 decreases gene expression in many ways, including interfering with a transcription factor, interfering with RNA Pol II, therefore inhibiting transcription, interfering with mRNA splicing, interfering with epigenetic regulation leading to gene silencing and competing with miRNAs, and preventing mRNA transcriptions. Figure 1 created with BioRender.com (Accessed on 27 December 2023).

Metastasis-associated lung adenocarcinoma transcript-1 (MALAT-1) is a long non-coding intergenic RNA of 12,820 bases on chr11q13. It is initially transcribed as a precursor transcript, followed by enzymatic processing by RNase P to form the mature long non-coding RNA [1,2]. A triple helical structure at the 3′ end stabilizes the structure of MALAT-1 and compensates for the missing poly-A tail. It regulates gene expression by various mechanisms, including modulating gene transcription through the repression of promoters of target genes, regulating RNA-binding proteins or activating mesenchymal transcription factors, modifying the chromatin, and regulating post-transcriptional processing (Figure 1) [3,4,5,6]. In addition, it is involved in DNA repair and cell death [7]. Targeting MALAT-1 induces DNA damage and sensitizes cancer to chemotherapy treatment [8]. 

**Figure 2:**
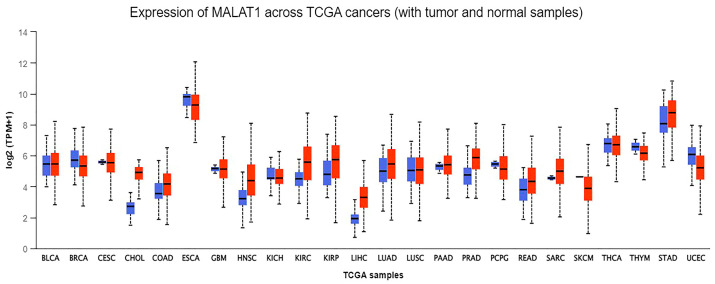
Depicts the comparison of MALAT-1 gene expression. MALAT-1 gene expression between tumor samples (denoted in red color) and non-cancerous samples (blue color) across different cancers through the UALCAN database online portal (https://ualcan.path.uab.edu/) (Accessed on 26 December 2023). The tumor sample shows high expression in various tumors such as esophageal carcinoma, CC, HCC, sarcoma, and melanoma compared to normal patients’ samples.

MALAT-1 induces cancer proliferation, invasion, migration, and metastasis to distant sites. Recently, several studies highlighted the immunomodulatory role of MALAT-1 and how it can enable cancer cells to escape immune surveillance by exerting an immunosuppressive effect and regulating the expression of several molecules associated with the tumor microenvironment [9,10]. In the triple-negative breast cancer (TNBC) cell model, MALAT-1 knockdown results in a marked induction in MHC class I chain-related proteins A/B expression and the repression of the checkpoint molecules PD-L1 and B7-H4 [11]. In addition, MALAT1 also modulates its suppressive effect by negatively modulating Myeloid-derived suppressor cells (MDSCs) and decreasing peripheral blood mononuclear cells (PBMCs) in cancer patients [12]. Indeed, MALAT-1 knockdown using MALAT-1 antisense oligonucleotides (ASO) in an immune-competent mouse model results in a decrease in MDSC as well as immunosuppressive tumor-associated macrophages (TAM). In contrast, an increase in cytotoxic CD8^+^ T cells was also observed, which opened new avenues in understanding the conspicuous role of MALAT-1 in modulating carcinogenesis [13]. Various studies have reported MALAT-1 overexpression in numerous cancers such as esophageal squamous cell carcinoma (ESCC), gastric cancer (GC), non-small cell lung cancer (NSCLC), colorectal cancer (CRC), pancreatic cancer, breast cancer (BC), and hepatocellular carcinoma (HCC) [14,15,16,17,18,19,20,21,22,23,24]. Using the online portal of the University of Alabama at Birmingham (UALCAN) (https://ualcan.path.uab.edu/) (Accessed on 26 December 2023), we analyzed MALAT-1 gene expression across different cancers using data from The Cancer Genome Atlas (TCGA) [25,26]. The expression levels of MALAT-1 between samples of different cancers and normal patients show that high expression of MALAT-1 is a prevalent event in various tumors such as ESCC, HCC, cholangiocarcinoma, cervical cancer (CC), sarcoma, and melanoma (Figure 2). Moreover, plausible differences in the expression of MALAT-1 between normal and cancerous samples were observed in other cancers, such as bladder carcinoma, thyroid carcinoma, and stomach adenocarcinoma (Figure 2). Contradictory to the disparity in MALAT-1 expression between normal and cancerous samples that are generally observed, samples having approximately the same MALAT-1 expression levels also exist. This ambiguity implies that MALAT-1 has a pleiotropic effect in cancer cells. 

MALAT-1 is a promising diagnostic marker for detecting endometrial, breast, NSCLC, bladder, and nasopharyngeal carcinoma (NPC) [27,28,29,30,31]. However, there is a discrepancy in reporting the diagnostic accuracy of MALAT-1 [29,30,31]. A pooled analysis including 17 studies with 3255 subjects demonstrated that MALAT-1 exhibits moderate accuracy in detecting and diagnosing cancer, and it was strongly associated with the metastasis of early-stage NSCLC [32]. A wealth of evidence has revealed that the role of MALAT-1 is pivotal in modulating EMT, driving cells to become cancer stem cells (CSCs) or acquire stem cell–like properties, develop chemoresistance, and metastasize to distant places in the body [17,33,34,35,36]. Albeit the role of MALAT-1 in cancer has been extensively studied, molecular mechanisms that regulate MALAT-1 are scarcely reported. Several reports examined the expression level of MALAT-1 and its significance as a diagnostic, prognostic, and, recently, as a novel drug target. However, the underlying mechanisms that induce MALAT-1 to play its role are still largely unknown. 

In EMT, epithelial cells display migratory and invasive features and become mesenchymal cells via the downregulation of E-cadherin, desmosomes, and claudin, and the upregulation of mesenchymal markers such as N-cadherin, fibronectin, and vimentin [37,38,39,40,41,42]. EMT increases the invasiveness and plasticity of cancer cells, which results in cancer cell dissemination to distant sites through the basement membrane, thereby inducing metastasis. Tumor-associated stroma upregulates the expression of various growth factors such as PDGF, EGF, HGF, and TGF-β, which in turn induces the activation of a series of transcription factors, including SNAI1, Slug, Twist, Zinc finger E-box binding homeobox1 (ZEB1), Goosecoid, and FOXC2, consequently initiating the EMT process [43,44,45,46,47,48]. Several reports demonstrate that EMT is crucial in stimulating cancer progression and metastasis and acquiring drug-resistant properties by modulating alternative cell signaling pathways [49,50,51,52,53]. Essential player proteins like Akt, ERK, MAPK, PI3K, β-catenin, and SMAD are essential in modulating EMT by central cell-signaling pathways [54]. Furthermore, microRNAs also have a crucial role in the cellular signaling circuitry that controls the EMT process. MALAT-1 serves as a competitive endogenous RNA (ceRNA) for tumor-suppressive microRNA and consequently downregulates their gene expression. 

This review will dissect the mechanisms by which MALAT-1 modulates EMT to enhance cancer proliferation, metastasis, stemness, and chemoresistance (Table 1). We will also thoroughly discuss the interaction between MALAT-1 and microRNA (Table 2). Different signaling pathways will also be explored. In this review, we provide an overview of the interplay between MALAT-1 and EMT and shed light on the importance of MALAT-1 in enabling cancer invasiveness, metastasis, stemness, and chemoresistance through several mechanisms. 

## 2. Methodology for Searching the Literature and Study Selection Criteria

In this review, the literature search was conducted using different controlled search terms with several conjunctions: (NEAT2 [TIAB] OR “metastasis-associated lung adenocarcinoma transcript 1” [TIAB] OR “metastasis-associated lung adenocarcinoma transcript-1” [TIAB] OR MALAT-1 [TIAB]) AND (“Epithelial-Mesenchymal Transition” [TIAB] OR “Epithelial-Mesenchymal Transitions” [TIAB] OR “Epithelial-Mesenchymal Transformation” [TIAB] OR “Epithelial-Mesenchymal Transformation” [TIAB] OR “Epithelial-Mesenchymal Transformations” [TIAB] OR EMT [TIAB]). Our search was executed using several databases, including PubMed, Web of Science, and Scopus. Associated citations of every article were examined. We included all articles published in English that discussed the mechanistic role of MALAT-1 in modulating EMT in different cancers. 

## 3. MALAT-1 Modulates EMT and Promotes Cancer Metastasis, Stemness, and Chemoresistance

### 3.1. MALAT-1 Induces Cancer Progression and Metastasis by Modulating EMT

The overexpression of MALAT-1 enhances the ability of tumor cells to migrate, invade, metastasize, and escape the cytotoxic effect of chemotherapy. One study reported that MALAT-1 inhibition decreases the progression and metastasis of CC in vitro and in vivo [56]. MALAT-1 knockdown upregulates the epithelial markers and downregulates the mesenchymal markers, contributing to an active EMT process [56]. In addition, the EMT regulator SNAI1 was markedly downregulated, indicating that SNAI1 regulates the expression of E-cadherin in response to MALAT-1 knockdown [56]. 

Furthermore, MALAT-1 downregulation inhibits cancer cell proliferation, invasion, and metastasis in GC cells in vitro and in vivo. MALAT-1 silencing upregulates E-cadherin while downregulating vimentin and reverses the EMT process, indicating that MALAT-1 has a role in inducing GC cells to undergo EMT [58]. Jiao et al. [21] demonstrated that MALAT-1 was highly expressed in cell lines and tissue samples of pancreatic cancer, and its knockdown was shown to induce apoptosis and suppress cell migration and invasion in pancreatic cancer [21]. Furthermore, MALAT-1 knockdown increased the expression of the epithelial marker E-cadherin and decreased the expression of the mesenchymal markers N-cadherin, SNAI1, Slug, and metalloproteinases (MMP2 and MMP9), indicating that EMT was inhibited [21].

**Figure 3:**
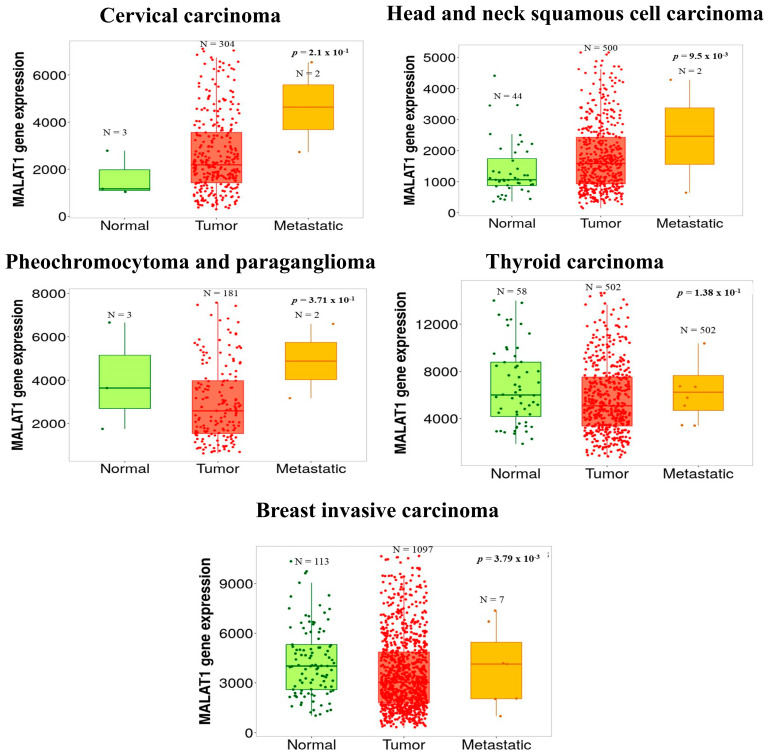
Illustrates the boxplot of the MALAT-1 expression. The analysis of MALAT-1 expression data through the online TNM plot portal (https://tnmplot.com/analysis/) (Accessed on 26 December 2023) compares the different expressions between normal, tumor, and metastatic samples in CC, HNSCC, pheochromocytoma and paraganglioma, thyroid carcinoma, and breast invasive carcinoma. Our results revealed that MALAT-1 expression is highly upregulated in metastatic samples compared to the tumor and normal samples.

Additionally, in lung cancer, Shen et al. [17] demonstrated that MALAT-1 expression was markedly higher in lung tumor samples with brain metastasis than those without brain metastasis, indicating EMT in metastatic samples [17]. MALAT-1 silencing inhibited the invasion and metastasis of a highly invasive subline of brain-metastatic lung cancer cells in vitro and in vivo. Intriguingly, E-cadherin and vimentin expression increased in this study, concluding that MALAT-1 promoted brain metastasis of lung cancer cells by inducing EMT [17]. Furthermore, we analyzed MALAT-1 RNA-Seq expression data using the TNM plot online portal (https://tnmplot.com/analysis/) (Accessed on 26 December 2023) in normal, tumor, and metastatic samples of CC, head and neck squamous cell carcinoma (HNSCC), pheochromocytoma and paraganglioma, thyroid carcinoma, and breast invasive carcinoma (Figure 3) [60]. Indeed, the analysis illustrated that MALAT-1 expression is significantly higher in metastatic samples than in tumor and normal samples, affirming the fundamental role of MALAT-1 in inducing cancer metastasis. Intriguingly, the graph in Figure 3 shows a difference in the distribution between normal and tumor samples and a remarkably high expression in metastatic samples, implying that MALAT-1 plays an essential role in inducing metastasis. 

### 3.2. MALAT-1 Promotes Chemoresistance via Modulating EMT

Several studies have discussed the role of MALAT-1 in facilitating chemoresistance in cancer [33,34,35,59]. Li et al. [59] revealed that a higher level of MALAT-1 in glioblastoma cells is associated with drug resistance through the upregulation of ZEB1 [59]. Knockdown of MALAT-1 markedly enhanced the sensitivity of multi-resistant glioblastoma cells to Temozolomide (TMZ) by decreasing the expression level of the resistance genes MDR1, MRP5, and LRP1 as well as the mesenchymal markers ZEB1, α-SMA, and fibronectin. These results affirm the pivotal role of MALAT-1 in modulating chemoresistance in cancer [59].

Another study investigates the role of MALAT-1 in modulating chemoresistance in CRC. Xiong et al. [33] established Oxymatrine-resistant CRC cells and showed that chemo-resistant cell lines possess many characteristics associated with EMT. The cells lose their polarity, downregulate E-cadherin, and upregulate vimentin [33]. MALAT-1 was also shown to be highly expressed in Oxymatrine-resistant CRC cells. Therefore, MALAT-1 silencing resulted in a significant upregulation of epithelial markers and downregulation of mesenchymal markers. Hence, MALAT-1 knockdown may reverse the EMT process and reduce chemotherapy resistance [33].

Moreover, another study conducted by Wu et al. [34] illustrated that the expression of MALAT-1 was higher in Trastuzumab-resistant HER2^+^ cells and metastatic TNBC cells than in normal cells. MALAT-1 silencing was associated with a more robust response to Trastuzumab treatment and decreased cell proliferation and invasion [34]. The overexpression of MALAT-1 was correlated with higher expression of EMT markers. Thus, MALAT-1 was proposed to induce resistance to Cisplatin through EMT [35]. MALAT-1 silencing reduces Cisplatin resistance in OSCC by reversing EMT and enhancing cell apoptosis [35]. In addition, another study reported that MALAT-1 knockdown in diffuse large B cell lymphoma cells resistant to Adriamycin induces autophagy-related death, accompanied by enhanced chemosensitivity [57]. Moreover, MALAT-1 knockdown promotes chemosensitivity to Gemcitabine in pancreatic cancer [36]. MALAT-1 tethered EZH2 to the CDH1 promoter and inhibited miR-218, resulting in resistance to oxaliplatin in CRC [61].

EMT acquisition has induced resistance to EGFR-TKIs in advanced NSCLC [62]. In addition, MALAT-1 has also been shown to induce resistance to the newly developed EGFR-TKI targeted therapy, Gefitinib [63]. In lung adenocarcinoma A549 cells that are resistant to Gefitinib, MALAT-1 expression was significantly higher than in normal cells [63]. MiR-200a has been reported to increase sensitivity to Gefitinib treatment in NSCLC [64]. MALAT-1 was shown to sponge miR-200a in Gefitinib-resistant A549 cells that inhibit EMT by targeting ZEB1 and ZEB2 [63,65]. Therefore, knockdown of MALAT-1 by shMALAT-1 significantly decreases cell proliferation and resistance to Gefitinib [63]. Collectively, the role of MALAT-1 in promoting chemoresistance is monumental and involves interference with several mechanisms that facilitate the development of resistance, including DNA damage and repair pathways, drug efflux, cell cycle, apoptosis, autophagy, stemness, and EMT reviewed extensively by Hou et al. [66]. We mainly focused on discussing the role of MALAT-1 in modulating EMT-induced chemoresistance. Therefore, MALAT-1 may be a potential target for pharmacological intervention to overcome chemotherapy resistance and enhance the response to the current treatment options. 

### 3.3. MALAT-1 Drives Cancer Cells toward More Stem Cell-like Features by Inducing EMT

Tumor heterogeneity is mainly caused by a minor subpopulation called CSCs, which is responsible for tumor plasticity, angiogenesis, invasion, and resistance to cancer treatment. CSC populations respond to the adjacent microenvironment by interacting with transcriptional, post-transcriptional, and metabolic factors [67,68]. Various lncRNAs have been associated with initiating, maintaining, and regulating CSCs, including HOTAIR, MALAT-1, HOTTIP, and H19 [69]. Jiao et al. [36] examined MALAT-1 expression in the pancreatic CSC population and showed that it was overexpressed in CSCs. Knockdown of MALAT-1 resulted in a decrease in the CD133^+^ subpopulation [36]. In addition, the induction of EMT using TGF-β increased the expression of MALAT-1 in the CD133^+^ subpopulation compared to the CD133^-^ subpopulation, indicating that MALAT-1 was upregulated in pancreatic CSCs. MALAT-1 knockdown decreases sphere formation, colony formation, and tumor size [36]. 

Furthermore, another study demonstrated that MALAT-1 promoted CSC-like phenotypes in the MCF7 cell line. MALAT-1 was markedly overexpressed in the CD133^+^ subpopulation compared to the rest of the MCF7 cells [55]. MALAT-1 silencing subsequently leads to a decrease in the CD133^+^ CSC population. In addition, the sphere formation rate, proliferation, colony formation, migration, and invasion of CSCs also decreased [55]. In conclusion, MALAT-1 promotes cell proliferation, migration, invasion, metastasis, and chemoresistance by promoting EMT, which may give rise to CSCs. However, further studies are required to decipher the mechanisms underlying inducing a CSC-like phenotype. 

## 4. MALAT-1 Regulates EMT by Competitively Inhibiting microRNAs, Enabling Cancer Invasion, Metastasis, and Chemoresistance

Non-coding RNAs (ncRNAs) are roughly divided into small ncRNAs fewer than 200 nucleotides long and long ncRNAs with more than 200. The category of small ncRNAs can be further divided into microRNAs (miRNAs), small interfering RNAs (siRNAs), and PIWI-interacting RNAs (piRNAs) [70]. A myriad of interactions has been shown to exist among small ncRNAs and long ncRNAs that regulate cell growth and survival [71]. MicroRNA (miRNA) is a group of non-coding RNAs with small nucleotide lengths ranging from 17–25 nucleotides. MiRNAs mainly regulate gene expression by directly interfering with mRNA [70]. Several miRNAs were deregulated in cancers such as glioblastoma, lung cancer, leukemia, BC, HCC, and thyroid carcinoma [72,73,74,75,76,77]. MiRNA profiling revealed that miRNAs are considered a signature for various types of cancer, such as lung adenocarcinoma, HCC, glioblastoma, papillary thyroid carcinoma, BC, and lymphocytic leukemia [72,73,74,75,76,77]. Therefore, profiling miRNA expression in different cancer types is extensively studied. Due to their dysregulation in different cancers, miRNAs are shown to be useful as a prognostic and diagnostic marker [78,79]. 

Many studies investigate the interplay between MALAT-1 and microRNAs. In normal cells, lncRNAs and miRNAs are famous for their crucial role in interfering with mRNA [80,81,82]. However, studies suggest that lncRNA and microRNA interactions are paramount in regulating cancer cells [83,84,85]. The study of the lncRNA–miRNA–mRNA axis has grown extensively in the last decade, paving the way for an advanced understanding of cancer biology [85]. The interaction between lncRNAs and miRNAs results in mRNA sequestering and degradation. A review article by Yoon et al. [86] extensively discusses the interaction between lncRNAs and miRNAs [86]. In these instances, we discuss the reports that addressed the interplay between lncRNAs and miRNAs and how this interaction influences the EMT process in cancer (Table 2).

**Table 2:** Summary of interaction between MALAT-1 and MicroRNAs modulating EMT, CSC properties, metastasis, and chemoresistance.

Cancer	MicroRNA	Target Gene	Effect	Mechanism	Cell Lines	In Vivo	References
CC	miR-202-3p	Periostin	Downregulate EMT and cancer metastasis	↑ E-cadherin, ↓ N-cadherin, ↓ vimentin, ↓ Periostin	H8, HeLa, and SiHa	-	[87]
CRC	miR-218	EZH2/CDH1	Downregulate EMT and chemoresistance	↑ E-cadherin	T29, SW480, SW620, and FHC	-	[61]
EEC	miR-200c	TGF-β	Downregulate EMT and cancer metastasis	↑ E-cadherin, ↓ ZEB1, ↓ N-cadherin, ↓ β-catenin, ↓ vimentin.	L-952, HEC-1-B, and JEC	BALB/c nude mice	[88]
EC	miR-1-3p	CORO1C/TPM3	Downregulate EMT and cancer metastasis	↑ E-cadherin, ↓ N-cadherin.	KYSE-510, and TE-7	BALB/c mice	[89]
HNSCC	miR-30a	TGF-β/STAT3	Downregulate EMT and cancer metastasis	↓ Twist, ↓ MMP2/9, ↓ STAT3, ↑ E-cadherin, ↓ N-cadherin, ↓ vimentin,	SCC15, SCC25, CAL27, and HaCaT	BALB/c-nu mice	[90]
HCC	miR-142-3p	SMAD5	Downregulate cancer cell growth, migration, and invasion	↓ vimentin, ↑ E-cadherin, ↓ SMAD5, ↓ Ki-67	Bel-7402, Huh-7, SMMC-7721, HL7702, and HepG2,	NOD/SCID mouse	[91]
	miR-125a-3p	FOXM1	Decrease cell proliferation, migration, and invasion	↓ FOXM1	Huh-6, HCCLM3, SK-HEP1, HuH-7, and PLC, L02	Female athymic nude mice	[92]
	miR-22	SNAI1	Downregulate EMT	↓ SNAI1, ↑ E-cadherin	HepG2, Hep3B, HuH7, and PLC/PRF5	BALB/c nude mice	[93]
Lung adenocarcinoma	miR-429	RhoA	inhibit cell growth and metastasis	↓ N-cadherin, ↑ E-cadherin, ↓ vimentin, ↓ Cyclin D1, ↓ MMP-9	BEAS-2B, HBE, A549, H1299, SPC-A-1, and PG49 HPAEpiC	-	[94]
	miR-204	Slug	Downregulate EMT and cancer metastasis	↑ E-cadherin, ↓ N-cadherin, ↓ vimentin, ↓ Slug	A549, H1299, H460, H446, and BEAS-2B	BALB/c-nu/nu mice	[95]
NPC	miR-124	Capn4	Downregulate EMT and cancer metastasis	↑ E-cadherin, ↓ N-cadherin, ↓ vimentin, ↓ Capn4	HNEpC, C666-1, HONE-1,5-8F, and CNE-2	-	[96]

### 4.1. MALAT-1 Regulates EMT by Competitively Inhibiting microRNAs in Hepatocellular Carcinoma

In HCC, Yu et al. [97] demonstrated that miR-142-3p is a potential MALAT-1-binding miRNA. The expression level of miR-142-3p in HCC tissues was lower than in adjacent normal tissues. MiR-142-3p has a tumor suppressor role in CC, suppressing proliferation and invasion capacities by inhibiting frizzled class receptor 7 (FZD7) [97]. MALAT-1 knockdown increased the expression of miR-142-3p in HCC cell lines. MiR-142-3p inhibited cell growth and promoted cell death by targeting SMAD5, a signal transducer protein belonging to the family SMAD [98]. SMAD5 works by activating different genes in the cell in response to signals from activated TGF-β. Hence, it induces cell proliferation, invasion, and EMT [98]. SMAD5 was more highly expressed in HCC cell lines than in adjacent cells. MALAT-1 modulates EMT by sponging miR-142-3p, resulting in the upregulation of SMAD5 expression and subsequently activating tumor invasion and metastasis [91]. 

Moreover, MALAT-1 binds and blocks miR-125a-3p, which targets FoxM1. FoxM1 is a transcription factor that increases cancer progression by inducing migration, invasion, metastasis, and EMT in cancer cells [99]. Knockdown of MALAT-1 upregulates the epithelial marker E-cadherin and downregulates the mesenchymal markers N-cadherin, vimentin, FoxM1, and SNAI1 [92]. MALAT-1 competitively binds to miR-125a-3p and activates FoxM1, inducing cancer proliferation and invasion [92]. Furthermore, one study reports that MALAT-1 sponges the tumor suppressor miR-22 by interacting with zeste homolog 2 (EZH2) enhancers to suppress miR-22 and E-cadherin. MALAT-1 sponging of miR-22 increased the expression of SNAI1 and activated EMT [93]. 

### 4.2. MALAT-1 Regulates EMT via Competitively Inhibiting microRNAs in Lung Cancers

A study conducted by Xiao and his colleagues demonstrated that MALAT-1 knockdown in PG49 and A549 cells significantly inhibits cell proliferation [94]. In addition, the expression of EMT markers were downregulated, while the expression of epithelial markers were upregulated [94]. MiR-429 is a tumor suppressor, and its expression was inversely correlated with MALAT-1 expression in lung adenocarcinoma. RhoA is a member of the Rho family of small GTPases participating in several biological processes, such as cell morphology, cell polarity, and cell invasion in cancer [100,101,102]. MiR-429 has been shown to downregulate MALAT-1 by binding to the RhoA 3′-UTR [94]. Therefore, MALAT-1 competes with miR-429 and inhibits its effect on RhoA, increasing cell proliferation, migration, and invasion [94]. Another study concluded that MALAT-1 competitively binds to miR-204 and suppresses its inhibitory effect on Slug. Slug is a transcription factor that regulates EMT by binding to the E-box sequence in the E-cadherin promoter [103]. MALAT-1 modulates EMT by increasing the Slug level and enhancing cancer proliferation and metastasis [95]. 

Furthermore, Tang et al. [104] demonstrated that MALAT-1 negatively correlates with mir-206. The overexpression of mir-206 was associated with inhibiting cell growth, migration, invasion, and metastasis. Silencing MALAT-1 reduces cancer migration, invasion, and metastatic properties [104]. Wu et al. [105] demonstrated that MALAT-1 binds to Ago2 and forms a complex that competitively inhibits miR-124. MALAT-1 knockdown upregulates the expression of E-cadherin, downregulates the expression of vimentin, and increases miR-124, which enhances cell apoptosis. This indicates that MALAT-1 regulates EMT by working as a competitive endogenous RNA and increasing NSCLC, thereby increasing cancer invasion and metastasis [105]. 

### 4.3. MALAT-1 Regulates EMT by Competitively Inhibiting microRNAs in Genitourinary Cancers

In CC, Han et al. [106] elucidated that the expression level of periostin was significantly high in both CC tissue samples and cell lines. Periostin is essential in modulating cell adhesion and migration [106]. Periostin knockdown inhibits CC cell proliferation, growth, and EMT. MALAT-1 was shown to be positively correlated with periostin level. In contrast, miR-202-3p was negatively associated with periostin levels. MiR-202-3p decreased periostin expression by binding to its 3′-UTR [87]. MALAT-1 sequesters miR-202-3p and leads to markedly increased expression of periostin. MALAT-1 silencing results in the upregulation of epithelial markers and downregulation of EMT markers, suppressing cell proliferation, invasion, and metastasis. This concludes that MALAT-1 regulates EMT via the MALAT-1/miR-202-3p/periostin axis [87].

Moreover, in endometrioid endometrial carcinoma (EEC), Li et al. [88] examined the expression of MALAT-1 and miR-200c in EEC tissue samples and cell lines [88]. They found a negative correlation between MALAT-1 and miR-200c. MiR-200c is significantly upregulated, while MALAT-1 is downregulated in EEC tissues and cell lines. By inhibiting miR-200c, the MALAT-1 expression level was increased and vice versa. MALAT-1 sponges miR-200c by binding to the 3′-UTR sequence of MALAT-1 [88]. MiR-200c is a tumor suppressor that decreases EEC’s invasion and migration in vitro. Silencing of MALAT-1 resulted in the upregulation of miR-200c expression and downregulation of the EMT markers such as β-catenin, ZEB1, and N-cadherin, which inhibit cell migration and metastasis [88]. In addition, MALAT-1 knockdown inhibits the effect of growth factor β (TGF-β) in inducing EMT in EEC in vitro. These results suggest that MALAT1 modulates EMT via the MALAT1/miR-200c /TGF-β axis [88].

### 4.4. MALAT-1 Regulates EMT by Competitively Inhibiting microRNAs in GIT Cancers

In Esophageal cancer (EC), Li et al. [89] observed that MALAT-1 expression was significantly higher in tumor tissues than in normal tissues. In contrast, the miR-1-3p expression level was downregulated considerably [89]. Furthermore, MALAT-1 knockdown decreased EMT markers and increased miR-1-3p expression levels, inhibiting the downstream effector CORO1C/TPM3. Silencing miR-1-3p upregulates the expression levels of MALAT-1, TPM3, and CORO1C and restores cell viability, migration, and invasion abilities, indicating that MALAT-1 regulates EMT via the miR-1-3p/CORO1C/TPM3 axis [89]. Furthermore, Li et al. [61] elucidated that MALAT-1 is overexpressed while miR-218 is downregulated. MALAT-1 tethered EZH2 to the CDH1 promoter and inhibited miR-218, inducing EMT, metastasis, and drug resistance [61]. Yes-associated protein 1 (YAP1) has been shown to have an oncogenic role in several cancers [107]. YAP1 has been shown to induce MALAT-1 expression, which promotes the expression of Twist, Slug, and VEGFA by sponging miR-126-5p, indicating that MALAT-1 promotes tumor metastasis by modulating EMT in CRC [108].

### 4.5. MALAT-1 Regulates EMT by Competitively Inhibiting microRNAs in Head and Neck Cancers

In HNSCC, Wang et al. [90] treated cancer cells with TGF-β and observed increases in cell invasion and migration capacities by increasing N-cadherin, vimentin, and Twist while inhibiting E-cadherin [90]. The study also reported that the signal transducers and activators of transcription (STAT3) expression were significantly increased in HNSCC cell lines after treatment with TGF-β [90]. STAT3 is a transcriptional activator that binds to MALAT-1 and induces cell proliferation, invasion, and EMT [109]. In addition, there is a mutual expression between STAT3 and MALAT-1, as STAT3 binds to the promoter region and activates MALAT-1 in HNSCC [90]. The administration of TGF-β inhibitor decreases the EMT phenotype and STAT3 activation, showing that the upregulation of TGF-β promotes EMT and STAT3 activation. MALAT-1 knockdown led to an increased E-cadherin level. In contrast, the levels of N-cadherin, Twist, vimentin, MMP2, and MMP9 were decreased. Mir-30a exerts an antitumor effect by decreasing cell invasion and inhibiting EMT. MALAT-1 competitively inhibits mir-30a and enhances EMT, subsequently increasing cell proliferation, invasion, and metastasis by sponging mir-30a [90]. In NPC, Shi et al. [96] showed that MALAT-1 and Capn4 were overexpressed, whereas miR-124 expression was decreased [96]. Capn4 is a small subunit of the calpain regulatory system, which is overexpressed in cancer and serves as a tumor promoter [110]. The author demonstrates that MALAT-1 knockdown inhibits cell proliferation, invasion, and EMT. A dual-luciferase reporter assay showed that MALAT-1 competitively inhibited mir-124. MiR-124 is a tumor suppressor that binds to the 3′-UTR of Capn4 and inhibits its effect. MALAT-1 acts as an endogenous competitor of miR-124 by sponging miR-124. Therefore, this decreases miR-124-induced suppression of Capn4 [96]. Previous studies revealed the interplay between MALAT-1 and different microRNAs in regulating EMT and inducing cancer invasion and metastasis. However, further studies are needed to demonstrate the link between lncRNA–miRNA–mRNA and how they affect EMT. Additionally, the interplay between miRNA and lncRNA varies among different cancer types. Further studies would allow us to gain a clearer understanding of the role of MALAT-1 in promoting EMT.

## 5. MALAT-1 Promotes EMT via Modulation of Different Signaling Pathways

**Figure 4:**
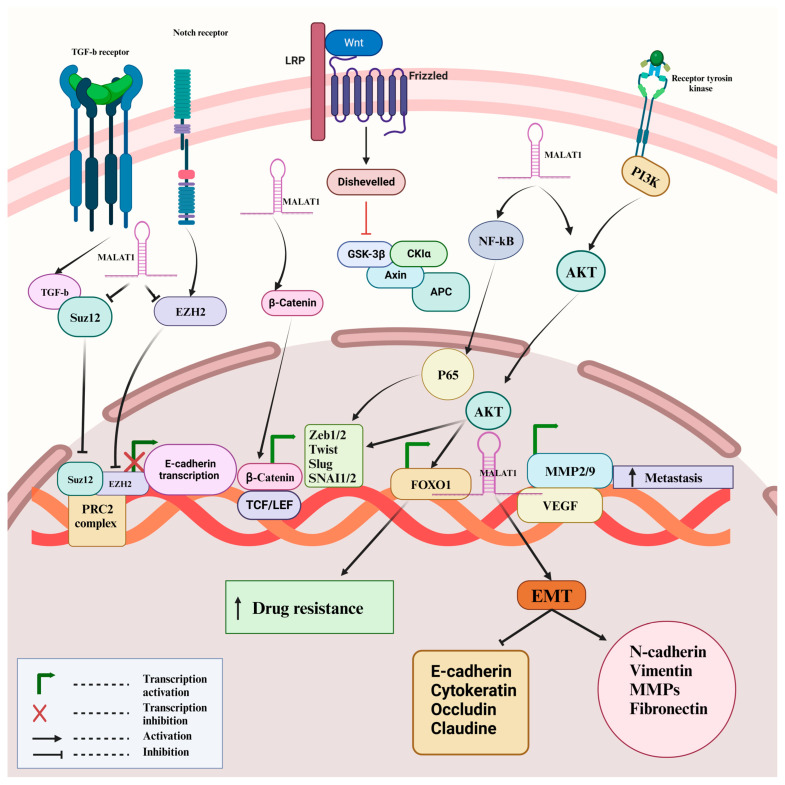
Emphasizes the different mechanisms implicated in MALAT-1 modulating EMT and metastasis. The PI3K/Akt pathway activates the protein kinase Akt to translocate to the nucleus and phosphorylates the transcription factor FOXO1, inhibiting EMT, thereby inducing chemoresistance. The activation of Wnt/β-catenin stimulates β-catenin migration to the nucleus and upregulates the expression of EMT transcription factors ZEB1/2, SNAI1/2, Twist1, and Slug, which in turn increase MALAT-1 gene expression. NF-kB activation leads to dislocation of the P65 subunit to the nucleus, which activates SNAI1/2, ZEB1/2, Twist1, and Slug, leading to the overexpression of MALAT-1 and activates VEGF and metalloproteinases MMP2 and MMP9, which in turn facilitate tumor invasion and metastasis. Notch and TGF-β activation bind to EZH2 and Suz12 and modulates post-transcription suppression of the epithelial markers. Figure 4 created with BioRender.com (Accessed on 27 December 2023).

As mentioned, MALAT-1 regulates EMT, metastasis, and chemoresistance in different cancers. Here, we discuss in detail the foremost pathways that intricately interact with MALAT-1 to modulate this process (Figure 4). 

### 5.1. PI3K/Akt Signaling Pathway

The phosphatidylinositol-3-kinase (PI3K/Akt) signaling pathway is paramount in controlling cancer cell growth, cell cycle, migration, invasion, and metastasis [111,112,113]. PI3K Activation leads to the phosphorylation of the downstream kinase Akt, which interacts with an intricate network inside the cell [114,115,116]. Studies show that MALAT-1 regulates PI3K/Akt in several cancers, such as osteosarcoma, gastric, breast, and cervical [34,117,118,119].

To study the role of MALAT-1 in regulating the PI3K/Akt signaling pathway, Wu et al. [34] examined the interaction between MALAT-1 and the PI3K/Akt signaling pathway in BC and showed that MALAT-1 induces EMT, promoting cell invasion, metastasis, and chemoresistance. The downregulation of MALAT-1 was associated with an enhanced response to chemotherapy, decreased cell growth, and invasion [34]. The EMT markers Slug, SNAI1, Twist, and Nanog were highly expressed in Trastuzumab-resistant HER2^+^ cell lines compared to their parental-resistant cell lines. However, these markers significantly decreased after MALAT-1 knockdown. MALAT-1 knockdown decreased the EMT transition phenotype and cell invasion in different types of BC, including TNBC, Trastuzumab-resistant HER2^+^ cells, and other subtypes [34]. The transcription factor FOXO1 modulated MALAT-1 in osteosarcoma [120]. It has been shown that FOXO1 modulates the effect of MALAT-1 by interacting with PI3K/Akt [34].

Paradoxically, Xu et al. [20] demonstrated that the level of MALAT-1 was significantly under-expressed in BC tissues than in adjacent normal tissues, indicating that MALAT-1 may act as a tumor suppressor instead of being identified as an oncogene [20]. MALAT-1 increased morphological features such as the spindle-like shape of the cell, invasiveness, and fibroblastic characteristics. EMT marker cadherin 2 (CDH2) was significantly upregulated in BC cell lines, while CDH1 was downregulated [20]. Furthermore, N-cadherin was significantly increased, and E-cadherin was decreased. In addition, knockdown of MALAT-1 markedly increased the expression levels of phosphorylated Akt (pS473) compared to control cells. These results conclude that MALAT-1 may regulate EMT through the PI3K/Akt pathway [20]. 

Furthermore, a study by Wang et al. [121] on cholangiocarcinoma concluded that MALAT-1 expression was significantly high in cholangiocarcinoma cells and tissue samples. MALAT-1 silencing inhibited cell viability, migration, invasion, and EMT. The epithelial marker E-cadherin was significantly increased, while the EMT marker vimentin was decreased [121]. This indicates that MALAT-1 promotes cholangiocarcinoma cell invasion and metastasis by modulating EMT-related proteins [121]. Silencing MALAT-1 downregulates the phosphorylated level of PI3Kand Akt, while the total protein level remains the same, indicating that MALAT-1 may modulate cholangiocarcinoma cell migration and invasion by activating the PI3K/Akt signaling pathway [121]. 

MALAT-1 knockdown inhibited cell growth and metastasis by inhibiting EMT in ovarian cancer. N-cadherin, vimentin, and SNAI1 were downregulated compared to E-cadherin [122]. The Akt protein expression level was higher than its phosphorylated form, confirming that the total protein level was not affected; meanwhile, p-Akt was significantly reduced, indicating that MALAT-1 silencing modulated EMT by downregulating the PI3K/Akt signaling pathway in ovarian cancer [122]. One study reports that in OSCC, overexpressed MALAT-1 activates the PI3K/Akt/mTOR signaling pathway, decreasing cell sensitivity to the chemotherapeutic agent Cisplatin [35]. 

### 5.2. Wnt/β-Catenin Signaling Pathway

In OSCC, MALAT-1 is overexpressed, and its silencing induces E-cadherin while decreasing N-cadherin and Vimentin expression. The expression of transcription factors such as ZEB1, Twist-1, and Slug was also decreased. MALAT-1 silencing decreased metastasis in vitro by suppressing the expression of MMP2, MMP9, and VEGF [123]. In addition, the authors also showed that MALAT-1 modulates the β-catenin and NF-κB signaling pathways. 

The downregulation of MALAT-1 decreased the expression of β-catenin, phosphorylated β-catenin, NF-κB-p65 subunit, and the activated form of NF-κB in both the nucleus and cytoplasm. This concludes that MALAT-1 modulates EMT by interacting with the β-catenin/NF-κB signaling pathway [123]. Furthermore, in bladder cancer, the knockdown of MALAT-1 markedly reduced β-catenin accumulation in the nucleus [124]. One study reported that in HCC, targeting MALAT-1 via shRNAs downregulates the expression of MALAT-1 and Wnt, indicating that MALAT-1 induces cell stemness and decreases differentiation through the Wnt pathway [125]. 

Another study reports that gallic acid decreases the expression of MALAT-1 in HCC cells. Gallic acid attenuated cell invasion and metastasis by inhibiting EMT [126]. An immunoblot assay revealed that upon treating HCC cells with gallic acid, the expression level of E-cadherin increased; in contrast, the expression levels of EMT markers N-cadherin, fibronectin, and vimentin along with the EMT transcription factors Twist, SNAI, and Zeb decreased [126]. Moreover, gallic acid inhibits the expression level of β-catenin at both mRNA and protein levels and many downstream effectors such as CCND1, VEGF, survivin, and Oct3/4. MALAT-1 has been shown to reverse the effect of gallic acid on β-catenin expression and localization, indicating that MALAT-1 mediates gallic acid’s inhibition of EMT via the Wnt/β-catenin pathway [126]. 

### 5.3. Other Signaling Pathways

One study on Kazakh’s esophageal squamous cell carcinoma investigated the interaction between MALAT-1 and TGF-β1. It showed that the combination of MALAT-1 knockdown and the TGF-β1 inhibitor significantly upregulates the expression level of E-cadherin compared to using TGF-β1 inhibitor alone, indicating that MALAT-1 inhibited TGF-β1-induced EMT [127]. Yu et al. [128] investigated the role of TGF-β1 in inducing MALAT-1 overexpression and EMT in bladder cancer. TGF-β1 treatment upregulated the EMT-related markers N-cadherin and fibronectin and downregulated the epithelial marker E-cadherin [128]. Upon knocking down MALAT-1, the expression of the epithelial-related marker E-cadherin was enhanced in bladder cancer. The authors also demonstrated that MALAT-1 inhibits E-cadherin epigenetically by binding to Suz12, inhibiting the effect of TGF-β1 in inducing EMT through the MALAT-1/Suz12 pathway [128].

Mingjiu et al. [129] showed that MALAT-1 expression was upregulated in EC cell lines. MALAT-1 knockdown inhibits cell proliferation, migration, invasion, and metastasis [129]. The expression levels of EZH2, Notch1, Hes1, MMP9, and vimentin decreased while the expression level of E-cadherin significantly increased. EC cell lines were co-transfected with shMALAT-1 and pcDNA3. The expression levels of EZH2, Notch1, Hes1, MMP9, and vimentin proteins were recovered, indicating the role of MALAT-1 in regulating EMT by modulating the EZH2–Notch1 signaling pathway [129]. More studies are warranted to decipher the direct interaction between MALAT-1 and different signaling pathways and whether dual inhibition will provide therapeutic advantages in preclinical models. 

## 6. Drug Targeting 

MALAT-1 has been shown to work as an oncogene and increase cancer invasiveness and metastasis. Therefore, MALAT-1 has tremendous potential as a candidate for therapeutic intervention. In general, several approaches have been exploited to target lncRNAs, including small molecules, deoxy ribozymes, ribozymes, nanobodies, small interfering RNAs (siRNAs), short hairpin RNAs (shRNAs), RNA decoys, antisense oligonucleotides (ASOs), aptamers, and mixmers [130]. Despite its location inside the nucleus, which renders its selective targeting problematic, multiple methods to target MALAT-1 in a preclinical model have been used, including shRNAs, ASO, and small molecules [130]. Chang et al. [125] demonstrated that shRNA-mediated knockdown of MALAT-1 resulted in downregulating the mesenchymal markers vimentin and Twist1. In addition, β-catenin and c-Myc were downregulated, indicating that targeting MALAT-1 suppresses HCC stemness and metastatic properties [125]. In osteosarcoma, small interfering RNAs target MALAT-1 to reduce tumor size, suppress tumor proliferation capability, and abrogate its pro-angiogenic effects [131]. Another approach used nanostructure conjugate ASO to increase nuclear delivery and ASO stability. One study used ASO and the nucleus-targeting TAT peptide conjugated to Au nanoparticles (ASO-Au-TAT NPs) to target MALAT-1 in lung cancer. The study shows that ASO-Au-TAT NPs decrease MALAT-1 expression and inhibit apoptosis [132]. Drug targeting MALAT-1 is still not a fully discovered area due to the difficulty of targeting and possible interactions with nonspecific RNA.

Natural products can also be used to target MALAT-1. In prostatic cancer, Lu et al. [116] have elucidated that Quercetin-treated prostatic cancer cell lines inhibit MALAT-1 in a dose- and time-dependent manner. Quercetin inhibited cell growth, invasion, and migration through MALAT-1 inhibition [116]. Quercetin markedly inhibited the phosphorylation of Akt, increased the expression of E-cadherin, and decreased N-cadherin [116]. Quercetin treatment inhibited EMT through MALAT-1 modulation of the PI3K/Akt signaling pathway [116]. Chen et al. [133] demonstrated that the pentacyclic triterpene Betulinic acid (BA) suppresses the expression level of the MALAT-1 gene and induces autophagy apoptosis in HCC [133]. In addition, the natural polyphenolic phytoalexin drug Resveratrol and its derivatives (3,5,4′-trimethoxystilbene and triacetyl Resveratrol) were shown to inhibit the expression of MALAT-1 and the EMT process in several cancers [134,135,136]. Yang et al. [52] revealed that the natural product Resveratrol extracted from several plants exhibits an anticancer effect by targeting MALAT-1 [52]. Resveratrol decreases the expression level of MALAT-1 and the EMT marker vimentin in GC cell lines. Resveratrol inhibits the metastatic and invasion capabilities of GC mediated by MALAT-1, confirming the genuine implication of MALAT-1 in modulating EMT in different cancers [52]. Moreover, in cutaneous squamous cell carcinoma (CSCC), the flavonoid dihydromyricetin (DHM) decreases the expression of MALAT-1. The overexpression of MALAT-1 inhibits transcription factor EB (TFEB) and subsequently inhibits autophagy in CSCC [137]. Natural products seem promising in targeting MALAT-1, devoid of the complexity associated with ASOs and siRNAs. However, more investigation is warranted to decipher how different natural products inhibit MALAT-1.

In silico studies have shown that RNA can target specific druggable pockets resulting from the higher-order structure and resembling proteins, paving the way for further studies in vitro and in vivo [138]. However, discussing drug docking is beyond the scope of this review. We will summarize the promising in silico small molecules targeting the triple helix or element for nuclear expression (ENE) of MALAT-1. Abulwerdi et al. [139] used the compound microarray strategy and found that small molecules can target the ‘3 terminal stability ENE that constitute the helical configuration responsible for MALAT-1 stability and function. The authors show that two molecules with benzimidazole and imidazole scaffolds have a promising result in targeting MALAT-1 [139].

Moreover, they used an organoid model of mammary cancer to investigate the biological role of these molecules. These compounds significantly decrease MALAT-1 and organoid branching [139]. Another study used silico models to test in silico compounds against the MALAT-1 triple helix and ENE core and found that 12 MALAT-1 targeting compounds (MTC) can target MALAT-1 at different binding sites [138]. In addition, another study revealed that the innovative small molecule of diphenylfuran scaffold binds to the MALAT-1 triple helix, disturbing the stability of MALAT-1 and inducing degradation in vitro [140]. The novel bifacial peptide nucleic acids (bPNAs) are a polypeptide oligomer, which resembles DNA and RNA, which binds to the U-rich internal loop in the ENE and disrupts the binding of MALAT-1′ oligo-A tail to the U-rich internal loop, exposing MALAT-1 to exonuclease activity and subsequently decreasing its expression [141]. In addition, several innovative CRISPR-Cas9 systems have been recently developed to target MALAT-1 [142,143]. Together, these results open a new era for targeting RNA implicated in a wide range of diseases by designing small-molecule probes targeting specific binding sites found in the higher structure of RNA. However, further in vitro and in vivo studies are warranted. 

## 7. Conclusions and Future Prospective 

Multiple lines of evidence insinuate that MALAT-1 plays a crucial role in cancer by regulating EMT. It is significantly overexpressed in several cancer types and functions by enhancing cell proliferation, invasiveness, metastasis, and chemoresistance. The salient features of MALAT-1 include inducing EMT by activating many critical pathways in cancers, such as β-catenin, PI3K, Wnt, TGF-β, and Ezh2-Notch1. In addition, MALAT-1 competes with a wide range of different miRNAs and interferes with transcription factors that drive the EMT process, leading to increased cancer invasiveness and chemoresistance. MALAT-1 is a promising candidate for drug targeting as it possesses properties that render it efficiently targetable compared to complex pathways with intricate interactions within the cell. More studies are needed to elaborate on the role of MALAT-1 in modulating CSCs and targeting MALAT-1 in different cancer types using different strategies.

## Figures and Tables

**Table 1:** Various mechanisms of MALAT-1 modulate EMT, chemoresistance, and CSC properties in different cancers.

Cancer Type	Phenotype	Effect	Mechanism	Cell Lines	In Vivo	References
BC	Cancer stem cell-like properties	Modulate stem cell-like properties in BC	↓ CD133^+^, ↓ ALDH^+^, and ↓ Sox2	MCF7	-	[55]
CC	EMT	Inhibit invasion and metastasis	↑ E-cadherin, ↑ ZO-1, ↓ vimentin, ↓ β-catenin, and ↓ Snail1	H8, CC, CaSki, HeLa, SiHa	Female BALB/c nude mice	[56]
CRC	Chemoresistance	Reverse EMT and reverse Oxymatrine resistance	↑ E-cadherin, and ↑ vimentin	HT29, SW480, HT29 Oxymatrine resistant	-	[33]
Diffuse large B-cell lymphoma	Chemoresistance	Enhance drug sensitivity by inducing autophagy in DLBCL	↑ LC3-II, ↑ LC3-I, and ↓ p62	IM-9I, Ly3, Ly8, Pfeiffer, Farage, Raji, Daud, Ly10, Ly1	BALB/c-nu/nu nude mice	[57]
GC	EMT	Inhibit invasion and metastasis	↑ E-cadherin, and ↓ vimentin	SGC-7901, BGC823, AGS, MKN4, SGC7901M, SGC7901NM	Female nude mice	[58]
Glioblastoma	Chemoresistance	Increase sensitivity to Temozolomide	↓ MDR1, ↓ MRP5, ↓ LRP, ↓ ZEB, ↑ E-cadherin, ↑ ZO-1, ↓ SMA, and ↓ Fibronectin	U251, U87, U251/TMZ, U87/TMZ	Nude mouse	[59]
Lung cancer with brain metastases	EMT	Inhibit invasion and metastasis	↑ E-cadherin, and ↑ vimentin	H1915-L10, H1915-H10	Athymic BALB/c-nu/ nu mice	[17]
Oral squamous cell carcinoma (OSCC)	Chemoresistance	Reverse EMT and increase Cisplatin chemosensitivity	↓ p-PI3K, ↓ PI3K ↓ p-AKT, ↓ AKT, ↓ pm-TOR, ↓ mTOR, and ↑ E-cadherin	CAL-27, SCC-9	BALB/c nude mice	[35]
Pancreatic cancer	EMT	promote apoptosis, inhibit tumor invasion and migration	↑ p21, ↑ p53, ↓ CDC2, ↓ Snail, ↓ Slug, ↑ E-cadherin, ↓ N-cadherin, ↓ vimentin, ↓ MMP-2, and ↓ MMP-9	BxPC-3, AsPC-1, PANC-1, CFPAC-1, CAPAN-1, SW1990, HS-766T	-	[21]
	Cancer stem cells like phenotype and chemoresistance	Modulate stem cell-like phenotype and enhance drug sensitivity	↓ CD133^+^, and ↓ Sox2	AsPC-1, CFPAC-1,	BALB/c nude mice	[36]
TNBC	Chemoresistance	Reverse EMT and reverse Trastuzumab resistance	↓ Snail, ↓ Slug, ↓ Twist, ↓ Nanog	SKBR3, BT474, MCF7, T47D, MDA-MB231, MCF-12A, JIMT1	Female athymic nude mice	[34]

## Abbreviations

ASOs	Antisense oligonucleotides
CC	Cervical cancer
CDH1	Cadherin 1
CRC	Colorectal cancer
EEC	Endometrioid endometrial carcinoma
EMT	Epithelial to mesenchymal transitions
ESCC	Esophageal Squamous Cell Carcinoma
HCC	Hepatocellular carcinoma
HNSCC	Head and neck squamous cell carcinoma
lncRNA	Long non-coding RNA
MALAT-1	Metastasis-associated lung adenocarcinoma transcript 1
NEAT2	Nuclear enriched abundant transcript 2
NPC	Nasopharyngeal carcinoma
NSCLC	Non-small cell lung cancer
nt	Nucleotides
OSCC	Oral squamous cell carcinoma
TMZ	Temozolomide
